# Numerical analysis of the chemically reactive EMHD flow of a nanofluid past a bi-directional Riga plate influenced by velocity slips and convective boundary conditions

**DOI:** 10.1038/s41598-022-20256-x

**Published:** 2022-09-23

**Authors:** Ebrahem A. Algehyne, Amal F. Alharbi, Anwar Saeed, Abdullah Dawar, Poom Kumam, Ahmed M. Galal

**Affiliations:** 1 Department of Mathematics, Faculty of Science, University of Tabuk, P.O. Box 741, Tabuk, 71491 Saudi Arabia; 2 Nanotechnology Research Unit (NRU), University of Tabuk, Tabuk, 71491 Saudi Arabia; 3 Department of Mathematics, Faculty of Sciences, King Abdulaziz University, Jeddah, Saudi Arabia; 4 Department of Mathematics, Faculty of Science, University of Tabuk, Tabuk, Saudi Arabia; 5 Center of Excellence in Theoretical and Computational Science (TaCS-CoE), Science Laboratory Building, Faculty of Science, King Mongkut’s University of Technology Thonburi (KMUTT), 126 Pracha-Uthit Road, Bang Mod, Thung Khru, Bangkok, 10140 Thailand; 6 Department of Mathematics, Abdul Wali Khan University, Mardan, Khyber Pakhtunkhwa 23200 Pakistan; 7 Department of Medical Research, China Medical University Hospital, China Medical University, Taichung, 40402 Taiwan; 8 Department of Mechanical Engineering, College of Engineering in Wadi Alddawasir, Prince Sattam Bin Abdulaziz University, Al-Kharj, Saudi Arabia; 9 Production Engineering and Mechanical Design Department, Faculty of Engineering, Mansoura University, Mansoura, 35516 Egypt

**Keywords:** Engineering, Mathematics and computing

## Abstract

This report presents the three-dimensional electromagnetohydrodynamic flow of a zinc-oxide–water nanofluid past a bidirectional Riga plate with velocity slips and thermal and mass convection conditions. The Cattaneo–Christov heat and mas flux model, thermal radiation, chemical reaction and activation energy are considered to analyze the flow problem. The volume fraction of the ZnO nanoparticles is taken 6% in this analysis. An appropriate set of similarity variables is used to transform the partial differential equations into ordinary differential equations. During this process, some parameters are found and influences of these factors on the flow profiles are shown and discussed in detail. A numerical technique called NDSolve is considered for the solution of the nanofluid flow problem. The results showed that higher solid volume fraction and slip parameter have reduced velocities profiles and the increasing solid volume fraction and thermal Biot number have increased the temperature profile. Additionally, the concentration Biot number has increased the concentration profile. The modified Hartmann number has significantly increased the velocity profile. Dual impacts in velocity profiles along primary and secondary direction has been observed due to stretching ratio parameter. A comparison of current results has been carried with a fine agreement amongst current and established results.

## Introduction

Nowadays researchers have created numerous easier and less expensive methods to create nanoparticles of economically significant materials as a result of the special features of nanotechnology. A number of metal oxide nanoparticles are created with potential future uses. One of them is zinc oxide which is one of the best utilized at the nanoscale. ZnO is an inorganic substance having numerous applications at the industrial level such as capacitors, cerium, porcelain, glass, concretes, polymers, polyester, adhesives, and colors etc. ZnO is a semiconductor in nature and has various distinctive qualities, including strong room-temperature fluorescence, high mobility of electrons and superb transparency etc. Zinc oxide is crucial for both scientific and industrial applications due to its wide band gap and high excitonic binding energy.

In past few decades the rheological and thermal characteristics of nanoparticles base fluids have been captured more consideration due to their important applications at industrial level. Such fluids are termed as nanofluid and can be manufactured by suspending a single type of nanoparticles in pure fluids. It has been shown that such fluids have tremendous thermal conductivities due to which these fluids are using for coolant purposes such as, coolant of heat exchangers, nuclear reactors, electrical devices and coolant of auto engines etc. Choi and Eastman^1^ has presented the nano particulates to pure fluid for the enhancement its thermal conductivities. Ayub et al.^2^ have discussed electrically conducted nanoparticles-based fluid over a Riga plate and have revealed that flow of fluid has declined while thermal flow has supported by the augmenting values of nanoparticles volumetric fractions. Ramesh et al.^3^ have assessed the bioconvective Maxwell nanofluid flow past a Riga sheet by incorporating nonlinear thermal radiations and activation energy effects. It has highlighted by the authors that greater activated energy factor has enhanced the concentration of fluid. Ali et al.^4^ have introduced rotary transient nanofluid flow upon Riga plate by considering the effects of microbes, chemical reaction and non-Fourier thermal flux and noticed that the thermal curve rises with increment of thermophoresis and Brownian effects and have retarded with higher values of magnetic effects. Singh and Ghosh et al.^5^ provided a thorough numerical and experimental analysis, in order to assess the thermal functionality of the 60° and 30° Chevron plate geometries employing (MWCNT)/distilled water nanoliquid as refrigerant. Pal and Mandal^6^ have conducted melted heat transportation phenomenon for Sisko nanofluid flow upon stretching surface. Waqas et al.^7^ inspected numerically the bioconvective flow of nanofluid flow with impact of activated energy past a Riga surface and noticed that energy communication rate decayed by augmentation in Prandtl number. Singh et al.^8,9^ reported the computational and experimental study employing graphene oxide nanoliquids at various compositions and flow rates. The research revealed that utilising nanofluid caused a pressure losses and increased pouring energy, but improved thermal conduction, thermal efficiency, performance, and ultimate energy transfer index by 13%, 14%, 9%, and 10%, respectively. The dynamic viscosity and density of Al2O3/distilled water, (MWCNT)/DW, and graphene nano particulate nanoliquids was described by Singh and Ghosh^10,11^.

The Riga surface is an eminent actuator that comprises of ever-holding constant electrodes and magnet producing Lorentz forces, where such forces weaken swiftly at some distance from the surface known as Riga Plate. For its important applications at industrial level, researchers have been convinced to conduct numerous investigations for fluid flow upon Riga plates. Bhatti and Michaelides^12^ discussed the influences of activated energy on thermal bioconvective nanoparticles flow over a Riga plate and have revealed that higher values of Peclet and bioconvective Schmidt numbers have declined the profiles of microbes. Shafiq et al.^13^ have studied nanoliquid flow past a radiated Riga plate and have revealed that the augmenting values of radiated factor have enhanced the thermal profiles. Rasool et al.^14^ have investigated Marangoni based forced convection nanofluid flow past a Riga plate influenced by magnetic effects. It has shown in this study that effective Lorentz force has controlled the fluid motion and thermal flow. Mburu et al.^15^ have inspected numerically the generation of irreversibility and heat transmission for nanoparticles flow upon Riga plate. Pal and Mandal^16^ have inspected mixed convection electrically conducted nanofluid flow past a shrinking/stretching surface with heat source and viscous dissipation.

The collective deliberation of mass as well as thermal transportation has a considerable importance in industrial and engineering applications for instance, nuclear reactor coolant, thermal conduction in tissues, electronic devices coolant, food processing and air conditioning etc. The classical theories of Fourier^17^ and Fick^18^ have provided a base for heat and mass transportation problems. But since relaxation time are changing that has to affect thermal as well as concentration fields, so these two concepts were not more suitable for thermal and mass transportation problems. Cattaneo^19^ primarily has modified the theory of Fourier’s for thermal diffusions. This idea of Cattaneo was further modifies by Christov^20^ by exchanging time-based partial derivative with Oldroyd upper convection derivatives. Afterwards, a number of studies have been published in literature by considering the influence of heat and mass flux model of Cattaneo–Christov in the fluid flow models. Naseem et al.^21^ have inspected analytically the nanoliquid flow past a Riga plate using Cattaneo–Christov (CC) approach and have revealed that the thermal as well as concentration characteristics have been augmented with upsurge in relaxation parameters. Pal and Mandal et al.^22^ examined the magnetic flux in a permeable media under the impact of thermal radiation and varying thermal conductivity, to investigate the thermal performance of nanofluid over a stretched surface. Rasool and Wakif^23^ have inspected EMHD mixed convection flow of nanoparticles-based fluid towards vertically placed Riga plate using CC model. Their outcomes have been indicated that the escalating values of Hartman number have augmented the horizontal motion of fluid while thermal flow and concentration of nanoparticles have been reduced with higher values of relaxation time factor.

The smallest quantity of energy that is required for different compounds to commence a chemical reaction is termed as activation energy introduced first by Svante Arrhenius for instance, the energy required by a car to start its engine etc. Due to its important industrial and engineering applications many investigations have been conducted by various researchers to discuss the mass diffusions for fluid flow problems. Ali et al.^24^ have discussed bioconvective rotary motion of Maxwell nanoparticles over a Riga plate with influence of activation energy. It has established by the authors that the thermal flow rate has been augmented with upsurge in the values of Brownian and thermophoresis factors while concentration of fluid has been reduced by higher values of activation factor of energy. Shahid et al.^25^ have discussed experimentally the influence of activation energy nanofluid flow upon a porous surface and have determined the numerical outcomes for considered flow problem. Xia et al.^26^ discoursed the energy transmissions for nonlinear mixed convection nanofluid flow with influence of activation energy subject to multiple slip conditions at boundaries. Their outcomes have depicted that the thermal flow rate have been upsurge while fluid motion have been declined with higher values of Hartmann number and nanoparticles. Riaz et al.^27^ have studied the Cattaneo–Christov model for nonlinear convection nanofluid flow over an extending surface subject to activation energy and have revealed that concentration of fluid flow has been upsurge with growth in activation energy factor.

Thermal radiations play a pivotal role in transportation of heat. The effects of these radiations are pretty imperative in high thermal processes. Rehman et al.^28^ have deliberated the influence of thermal radiations upon fluid flow pattern over flat cylindrical surface and have concluded that thermal flow rate has been amplified with upsurge in the values of thermal radiation factor. Ahmad et al.^29^ have inspected the impacts of thermal radiations upon mass and heat transfers for fluid flowing in an infinite circular pipe. It has concluded by the authors of the work that fluid motion has retarded whereas the thermal flow rate has been enlarged with augmenting values of thermal radiation factors. Rehman et al.^30^ have conducted a comparative study for thermal transmissions of MHD Jeffery fluid flow with thermal radiations over cylinder shaped and plane sheets. Ashraf et al.^31^ have discussed thermally radiative nanofluid flow with influence of Darcy-Forchheimer model. Eid and Nafe^32^ inspected the variations in thermal conductivities and production of heat upon MHD nanofluid flow through porous medium and have determined that augmentation in thermal radiations and nanoparticles have augmented Nusselt number and while skin friction has retarded in this phenomenon. The readers can further study literature upon flow control with influence of thermal radiations in Refs.^33–38^.

After a careful analysis of the above-mentioned literature survey, we have observed that very less work based on the three-dimensional electromagnetohydrodynamic nanofluid flow past a bi-directional Riga plate with velocity slip and thermal and mass convective conditions is considered. Therefore, the authors have proposed this model. The flow contains the ZnO nanoparticles, which are suspended into water and analyzed through a bi-directional Riga plate with velocity slips and convection conditions. The consequences of Cattaneo–Christov heat and mas flux model, thermal radiation, chemical reaction and Arrhenius activation energy are studied. An appropriate set of variables is considered to convert the equations which governed the flow problem into dimension free form. A numerical technique called NDSolve is considered for the solution of the nanofluid flow problem. This work is composed of several sections which compiles the flow problem. “Problem formulation” section  represents the problem formulation. “Numerical solution” section  displays the numerical investigation of the present problem. “Results and discussion” section  shows the results and discussion of the flow problem. “Conclusion” section  is composed of concluding remarks.

## Problem formulation

**Figure 1:**
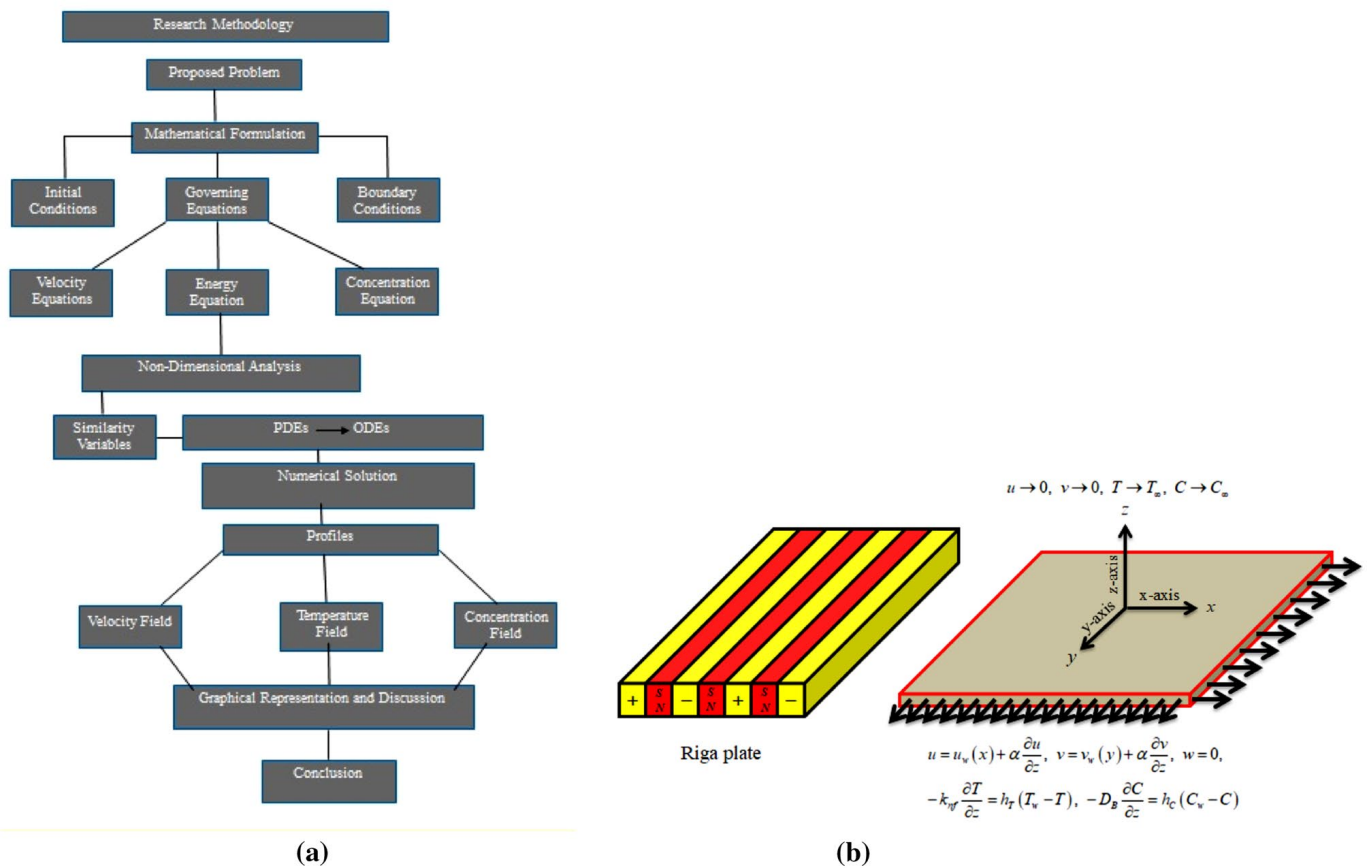
(**a**) Flow chart. (**b**) Flow geometry.

Consider the three dimensional EMHD flow of ${\mathrm{ZnO}}{-}{\mathrm{H}}_{2} {\mathrm{O}}$ nanofluid through a bi-directional Riga plate as shown in Fig. 1b. The stretching velocities along *x*- and *y*- axes are respectively described as $u_{w} \left( x \right) = ax$ and $v_{w} \left( x \right) = ay$, where $a$ and $b$ are constants such that both quantities are positive. The temperature and concentration of nanofluid at the surface of the plate are represented by $T_{w}$ and $C_{w}$, respectively while at free stream these values are depicted by $T_{\infty }$ and $C_{\infty }$, respectively such that $T_{w} > T_{\infty }$, $C_{w} > C_{\infty }$. Furthermore, the following assumptions are taken into consideration.

- Riga plate
- Non-Fick’s and non-Fourier model
- Arrhenius activation energy and thermal radiation
- Slip and convection conditions

With above stated suppositions the leading equations are^39–41^:

1 $$ \frac{\partial u}{{\partial x}} + \frac{\partial v}{{\partial y}} + \frac{\partial w}{{\partial z}} = 0, $$

2 $$ u\frac{\partial u}{{\partial x}} + v\frac{\partial u}{{\partial y}} + w\frac{\partial u}{{\partial z}} = \frac{{\mu_{nf} }}{{\rho_{nf} }}\frac{{\partial^{2} u}}{{\partial z^{2} }} + \frac{{\pi j_{0} M_{0} \left( x \right)}}{{8\rho_{nf} }}\exp \left( { - \frac{\pi }{{a_{0} }}z} \right), $$

3 $$ u\frac{\partial v}{{\partial x}} + v\frac{\partial v}{{\partial y}} + w\frac{\partial v}{{\partial z}} = \frac{{\mu_{nf} }}{{\rho_{nf} }}\frac{{\partial^{2} v}}{{\partial z^{2} }}, $$

4 $$ u\frac{\partial T}{{\partial x}} + v\frac{\partial T}{{\partial y}} + w\frac{\partial T}{{\partial z}} + \lambda_{E} \sigma_{E} = \frac{{k_{nf} }}{{\left( {\rho C_{p} } \right)_{nf} }}\frac{{\partial^{2} T}}{{\partial z^{2} }} - \frac{1}{{\left( {\rho C_{p} } \right)_{nf} }}\frac{{\partial q_{r} }}{\partial z}, $$

5 $$ u\frac{\partial C}{{\partial x}} + v\frac{\partial C}{{\partial y}} + w\frac{\partial C}{{\partial z}} + \lambda_{C} \sigma_{C} = D_{B} \frac{{\partial^{2} C}}{{\partial z^{2} }} - K_{r}^{2} \left( {C - C_{\infty } } \right)\left( {\frac{T}{{T_{\infty } }}} \right)^{m} \exp \left( {\frac{{ - E_{a} }}{KT}} \right), $$

where

6 $$ \sigma_{E} = \left\{ {\begin{array}{*{20}c} {u^{2} \frac{{\partial^{2} T}}{{\partial x^{2} }} + w^{2} \frac{{\partial^{2} T}}{{\partial z^{2} }} + v^{2} \frac{{\partial^{2} T}}{{\partial y^{2} }} + 2vu\frac{{\partial^{2} T}}{\partial x\partial y} + 2wu\frac{{\partial^{2} T}}{\partial x\partial z} + 2wv\frac{{\partial^{2} T}}{\partial y\partial z}} \\ { + \left( {u\frac{\partial u}{{\partial x}} + v\frac{\partial u}{{\partial y}} + w\frac{\partial u}{{\partial z}}} \right)\frac{\partial T}{{\partial x}} + \left( {u\frac{\partial v}{{\partial x}} + v\frac{\partial v}{{\partial y}} + w\frac{\partial v}{{\partial z}}} \right)\frac{\partial T}{{\partial y}} + \left( {u\frac{\partial w}{{\partial x}} + v\frac{\partial w}{{\partial y}} + w\frac{\partial w}{{\partial z}}} \right)\frac{\partial T}{{\partial z}}} \\ \end{array} } \right\}, $$

7 $$ \sigma_{C} = \left\{ {\begin{array}{*{20}c} {u^{2} \frac{{\partial^{2} C}}{{\partial x^{2} }} + v^{2} \frac{{\partial^{2} C}}{{\partial y^{2} }} + w^{2} \frac{{\partial^{2} C}}{{\partial z^{2} }} + 2uv\frac{{\partial^{2} C}}{\partial x\partial y} + 2uw\frac{{\partial^{2} C}}{\partial x\partial z} + 2vw\frac{{\partial^{2} C}}{\partial y\partial z}} \\ { + \left( {u\frac{\partial u}{{\partial x}} + w\frac{\partial u}{{\partial z}} + v\frac{\partial u}{{\partial y}}} \right)\frac{\partial C}{{\partial x}} + \left( {u\frac{\partial v}{{\partial x}} + w\frac{\partial v}{{\partial z}} + v\frac{\partial v}{{\partial y}}} \right)\frac{\partial C}{{\partial y}} + \left( {u\frac{\partial w}{{\partial x}} + w\frac{\partial w}{{\partial z}} + v\frac{\partial w}{{\partial y}}} \right)\frac{\partial C}{{\partial z}}} \\ \end{array} } \right\}. $$

The boundary conditions are:

8 $$ \begin{aligned} & u = u_{w} \left( x \right) + \alpha_{0} \frac{\partial u}{{\partial z}},\quad v = v_{w} \left( y \right) + \alpha_{0} \frac{\partial v}{{\partial z}},\quad w = 0, \\ & - k_{nf} \frac{\partial T}{{\partial z}} = h_{T} \left( {T_{w} - T} \right),\quad - D_{B} \frac{\partial C}{{\partial z}} = h_{C} \left( {C_{w} - C} \right)\quad at\quad z = 0, \\ & u \to 0,\quad v \to 0,\quad T \to T_{\infty } ,\quad C \to C_{\infty } \quad as\quad z \to \infty . \\ \end{aligned} $$

The radiative heat flux is defined as:

9 $$ q_{r} = - \frac{{4\sigma^{*} }}{{3k^{*} }}\frac{{\partial T^{4} }}{\partial z}, $$

Expending $T^{4}$ by Taylor series, we have:

10 $$ T^{4} \, \cong \,4T_{\infty }^{3} T - 3T_{\infty }^{4} + higher\,terms, $$

By ignoring higher terms, Eq. (10) can be reduced as:

11 $$ q_{r} = - \frac{{16\sigma^{*} T^{3} }}{{3k^{*} }}\frac{\partial T}{{\partial z}}, $$

Replacing Eq. (11) into (4), we have:

12 $$ u\frac{\partial T}{{\partial x}} + v \frac{\partial T}{{\partial y}} + w \frac{\partial T}{{\partial z}} + \lambda_{E} \sigma_{E} = \frac{{k_{nf} }}{{\left( {\rho C_{p} } \right)_{nf} }}\frac{{\partial^{2} T}}{{\partial z^{2} }} + \frac{{16\sigma^{*} T_{\infty }^{3} }}{{3k^{*} \left( {\rho C_{p} } \right)_{nf} }}\frac{{\partial^{2} T}}{{\partial z^{2} }}, $$

The thermophysical properties are defined as:

13 $$ \left\{ \begin{aligned} \mu_{nf} = \frac{{\mu_{f} }}{{\left( {1 - \Pi } \right)^{2.5} }}, \left( {\rho C_{p} } \right)_{nf} = \left( {1 - \Pi } \right)\left( {\rho C_{p} } \right)_{f} + \Pi \left( {\rho C_{p} } \right)_{n} , \hfill \\ \rho_{nf} = \left( {1 - \Pi } \right)\rho_{f} + \Pi \rho_{n} ,\quad k_{nf} = \frac{{2k_{f} + k_{n} - 2\Pi \left( {k_{f} - k_{n} } \right)}}{{2k_{f} + k_{n} + \Pi \left( {k_{f} - k_{n} } \right)}}k_{f} . \hfill \\ \end{aligned} \right\} $$

The thermophysical properties of pure fluid and nanoparticles are described numerically in Table 1.

**Table 1:** Numerical values of thermophysical properties of ${\mathrm{H}}_{2} {\mathrm{O}}$ and ${\mathrm{ZnO}}$^42–45^.

Base fluid and nanoparticle	$\rho \left[ {{\mathrm{Kg}}\,{\mathrm{m}}^{ - 3} } \right]$	$C_{p} \left[ {{\mathrm{J}}\,{\mathrm{Kg}}^{ - 1} \,{\mathrm{K}}^{ - 1} } \right]$	$k\left[ {{\mathrm{Wm}}^{ - 1} \,{\mathrm{K}}^{ - 1} } \right]$
${\mathrm{H}}_{2} {\mathrm{O}}$	997.1	4179	0.613
${\mathrm{ZnO}}$	5600	495.2	13

The set of appropriate variables are described as:

14 $$ u = axf^{\prime}\left( \xi \right),\quad w = - \sqrt {a\nu_{f} } \left( {f\left( \xi \right) + g\left( \xi \right)} \right),\quad v = ayg^{\prime}\left( \xi \right),\quad \theta \left( \xi \right) = \frac{{T - T_{\infty } }}{{T_{w} - T_{\infty } }},\quad \phi \left( \xi \right) = \frac{{C - C_{\infty } }}{{C_{w} - C_{\infty } }},\quad \xi = \sqrt {\frac{a}{{\nu_{f} }}} z. $$

Making use of Eq. (14) the leading equations are reduced as:

15 $$ \frac{{\mu_{nf} }}{{\mu_{f} }}f^{\prime\prime\prime}\left( \xi \right) + \frac{{\rho_{nf} }}{{\rho_{f} }}\left[ {\left( {f^{\prime\prime}\left( \xi \right)g\left( \xi \right) + f\left( \xi \right)} \right) - \left( {f^{\prime}\left( \xi \right)} \right)^{2} } \right] + H\exp \left( { - \beta \xi } \right) = 0, $$

16 $$ \frac{{\mu_{nf} }}{{\mu_{f} }}g^{\prime\prime\prime}\left( \xi \right) + \frac{{\rho_{nf} }}{{\rho_{f} }}\left[ {g^{\prime\prime}\left( \xi \right)\left( {f\left( \xi \right) + g\left( \xi \right)} \right) - \left( {g^{\prime}\left( \xi \right)} \right)^{2} } \right] = 0, $$

17 $$ \frac{1}{\Pr }\left( {\frac{{k_{nf} }}{{k_{f} }} + Rd} \right)\theta^{\prime\prime}\left( \xi \right) + \frac{{\left( {\rho C_{p} } \right)_{nf} }}{{\left( {\rho C_{p} } \right)_{f} }}\left[ {\left( {f\left( \xi \right) + g\left( \xi \right)} \right)\theta^{\prime}\left( \xi \right) - \delta_{T} \left\{ \begin{aligned} \left( {f\left( \xi \right) + g\left( \xi \right)} \right) \left( {f^{\prime}\left( \xi \right) + g^{\prime}\left( \xi \right)} \right) \hfill \\ \times \theta^{\prime}\left( \xi \right) +  \left( {f\left( \xi \right) + g\left( \xi \right)} \right)^{2} \theta^{\prime\prime}\left( \xi \right) \hfill \\ \end{aligned} \right\}} \right] = 0, $$

18 $$ \begin{aligned} & \frac{1}{Sc}\phi^{\prime\prime}\left( \xi \right) + \left( {f\left( \xi \right) + g\left( \xi \right)} \right)\phi^{\prime}\left( \xi \right) - K_{r} \left( {1 + \delta \theta \left( \xi \right)} \right)^{q} \exp \left( { - \frac{E}{1 + \delta \theta \left( \xi \right)}} \right)\phi \left( \xi \right) \\ & - \delta_{C} \left\{ {\left( {f^{\prime}\left( \xi \right) + g^{\prime}\left( \xi \right)} \right) \left( {f\left( \xi \right) + g\left( \xi \right)} \right)\phi^{\prime}\left( \xi \right) + \left( {f\left( \xi \right) + g \left( \xi \right)} \right)^{2}  \phi^{\prime\prime}\left( \xi \right)} \right\} = 0, \\ \end{aligned} $$

With conditions at boundaries as:

19 $$ \left\{ \begin{aligned} f^{\prime}\left( {\xi = 0} \right) = 1 + \alpha f^{\prime\prime}\left( {\xi = 0} \right),\quad f\left( {\xi = 0} \right) = 0, \hfill \\ g\left( {\xi = 0} \right) = 0,\quad g^{\prime}\left( {\xi = 0} \right) = \lambda + \alpha g^{\prime\prime}\left( {\xi = 0} \right), \hfill \\ \frac{{k_{nf} }}{{k_{f} }}\theta^{\prime}\left( {\xi = 0} \right) = Bi_{T} \left( {\theta \left( {\xi = 0} \right) - 1} \right),\quad \phi^{\prime}\left( {\xi = 0} \right) = Bi_{C} \left( {\phi \left( {\xi = 0} \right) - 1} \right), \hfill \\ f^{\prime}\left( {\xi \to \infty } \right) = 0,\quad g^{\prime}\left( {\xi \to \infty } \right) = 0,\quad \theta \left( {\xi \to \infty } \right) = 0,\quad \phi \left( {\xi \to \infty } \right) = 0. \hfill \\ \end{aligned} \right\} $$

Above the dimension-free parameters are mathematically described as:

20 $$ \left\{ \begin{aligned} H = \frac{{\pi M_{0} J_{0} }}{{8\rho_{f} au_{w} \left( x \right)}},\quad \beta = \frac{\pi }{{a_{0} }}\sqrt {\frac{{\nu_{f} }}{a}} ,\quad Sc = \frac{{\nu_{f} }}{{D_{B} }},\quad K_{r} = \frac{{k^{2} }}{a},\quad \lambda = \frac{b}{a}, \hfill \\ E = \frac{{E_{a} }}{{KT_{\infty } }},\quad \alpha = \alpha_{0} \sqrt {\frac{{\nu_{f} }}{a}} ,\quad Bi_{T} = \frac{{h_{T} }}{{k_{f} }}\sqrt {\frac{{\nu_{f} }}{a}} ,\quad Bi_{C} = \frac{{h_{C} }}{{D_{B} }}\sqrt {\frac{{\nu_{f} }}{a}} . \hfill \\ \end{aligned} \right\} $$

### Quantities Interest

The skin friction coefficients, local Nusselt and Sherwood numbers are described mathematically as:

21 $$ C_{fx} = \frac{{\tau_{wx} }}{{\rho_{f} \left( {u_{w} \left( x \right)} \right)^{2} }},\quad C_{fy} = \frac{{\tau_{wy} }}{{\rho_{f} \left( {v_{w} \left( x \right)} \right)^{2} }},\quad Nu_{x} = \frac{{xq_{w} }}{{k_{f} \left( {T_{w} - T_{\infty } } \right)}},\quad Sh_{x} = \frac{{xq_{m} }}{{D_{B} \left( {C_{w} - C_{\infty } } \right)}}, $$

where

22 $$ \tau_{wx} = \mu_{nf} \left. {\frac{\partial u}{{\partial z}}} \right|_{z = 0} ,\quad \tau_{wy} = \mu_{nf} \left. {\frac{\partial v}{{\partial z}}} \right|_{z = 0} ,\quad q_{w} = - k_{nf} \left. {\frac{\partial T}{{\partial z}}} \right|_{z = 0} + q_{r} ,\quad q_{w} = - D_{B} \left. {\frac{\partial C}{{\partial z}}} \right|_{z = 0} . $$

Using Eq. (14), the quantities of the interest are reduced as:

23 $$ C_{sx} = \frac{{\mu_{nf} }}{{\mu_{f} }}f^{\prime\prime}\left( 0 \right),\quad C_{sy} = \frac{{\mu_{nf} }}{{\mu_{f} }}g^{\prime\prime}\left( 0 \right),\quad \frac{{Nu_{x} }}{{\sqrt {{\mathrm{Re}}_{x} } }} = - \left( {\frac{{k_{nf} }}{{k_{f} }} + Rd} \right)\theta^{\prime}\left( 0 \right),\quad \frac{{Sh_{x} }}{{\sqrt {{\mathrm{Re}}_{x} } }} = - \phi^{\prime}\left( 0 \right), $$

Above ${\mathrm{Re}}_{x} = \frac{{u_{w} \left( x \right)x}}{{\nu_{f} }}$ and ${\mathrm{Re}}_{x} = \frac{{v_{w} \left( x \right)x}}{{\nu_{f} }}$ are local Reynolds numbers.

Also, $$C_{sx} = - \sqrt {{\mathrm{Re}}_{x} } C_{fx}$$ and $$C_{sy} = - \sqrt {{\mathrm{Re}}_{y} } C_{fy}$$.

## Numerical solution

When a physical phenomenon is modelled mathematically, it gave rise to nonlinear equation. Sometimes the nonlinearity of resultant equations is much higher. So, such equations are very difficult to solve analytically. In current problem the resultant Eqs. (15–18) are nonlinear for which NDsolve^46,47^ has used which is a numerical technique in Mathematica and is used to solve such problems efficiently.

## Results and discussion

**Figure 2:**
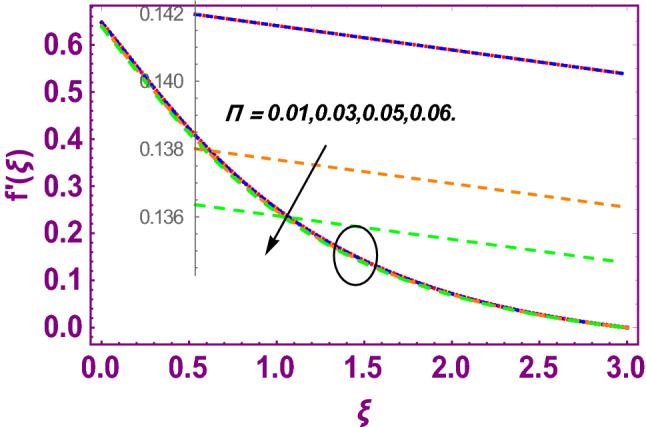
Effect of $\Pi$ on $f^{\prime}\left( \xi \right)$.

**Figure 3:**
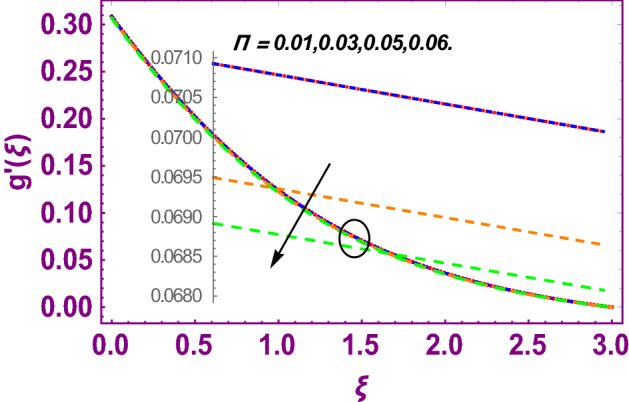
Effect of $\Pi$ on $g^{\prime}\left( \xi \right)$.

**Figure 4:**
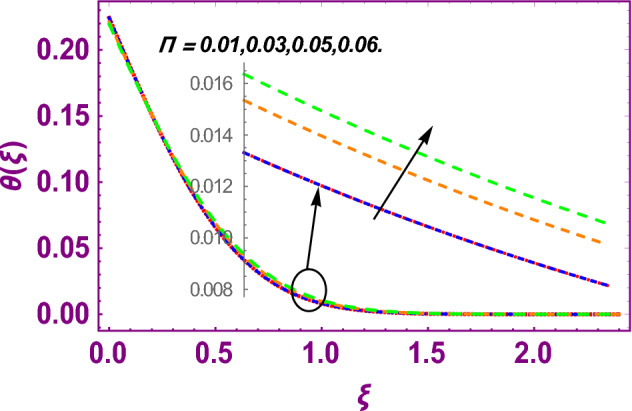
Effect of $\Pi$ on $\theta \left( \xi \right)$.

**Figure 5:**
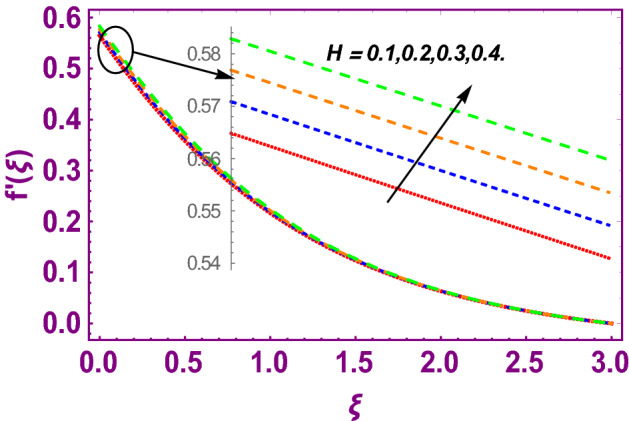
Effect of $H$ on $f^{\prime}\left( \xi \right)$.

**Figure 6:**
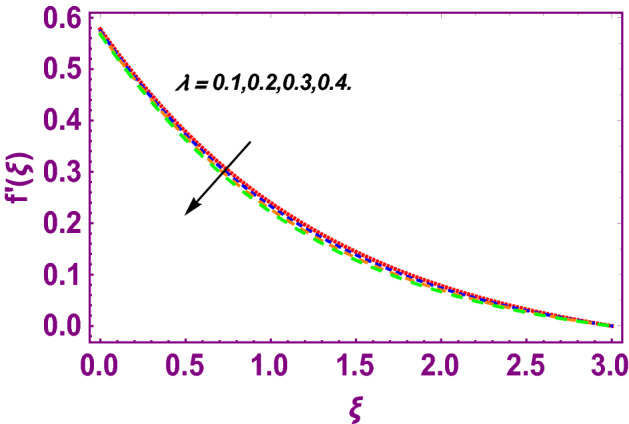
Effect of $\lambda$ on $f^{\prime}\left( \xi \right)$.

**Figure 7:**
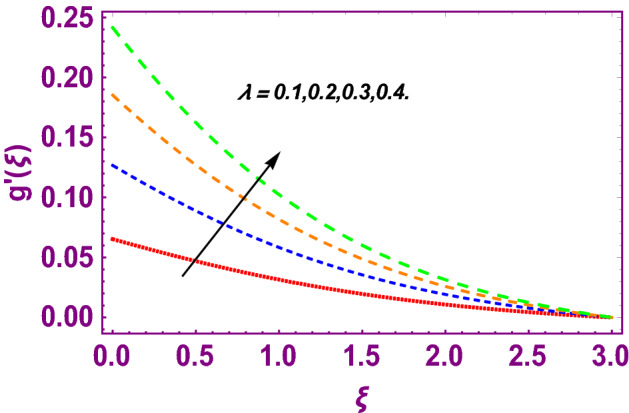
Effect of $\lambda$ on $g^{\prime}\left( \xi \right)$.

**Figure 8:**
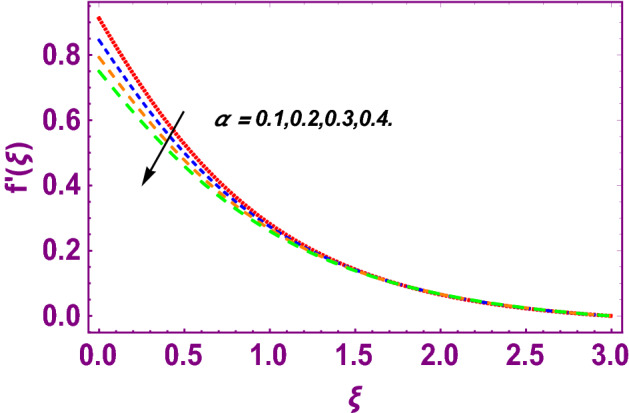
Effect of slip parameter on $f^{\prime}\left( \xi \right)$.

**Figure 9:**
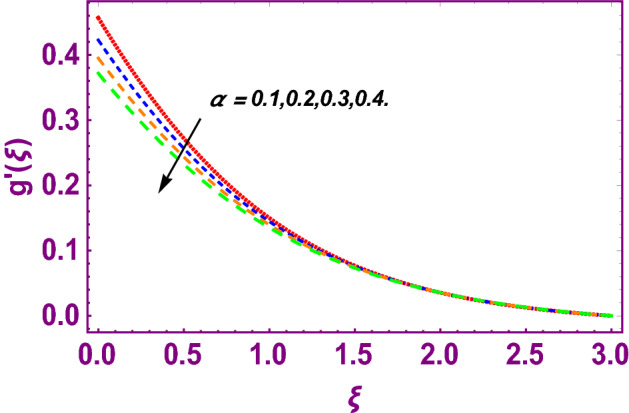
Effect of slip parameter on $g^{\prime}\left( \xi \right)$.

**Figure 10:**
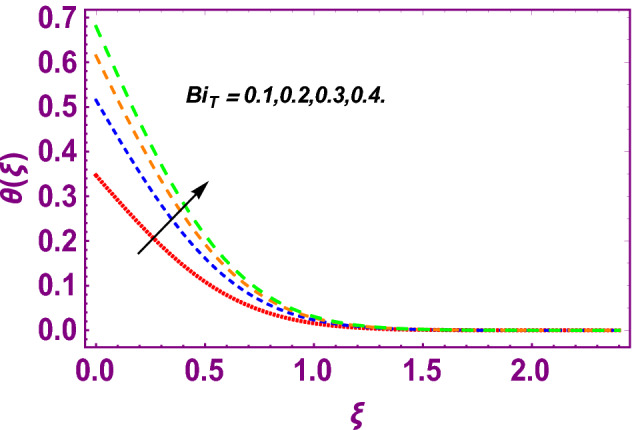
Influence of thermal Biot number upon $\theta \left( \xi \right)$.

**Figure 11:**
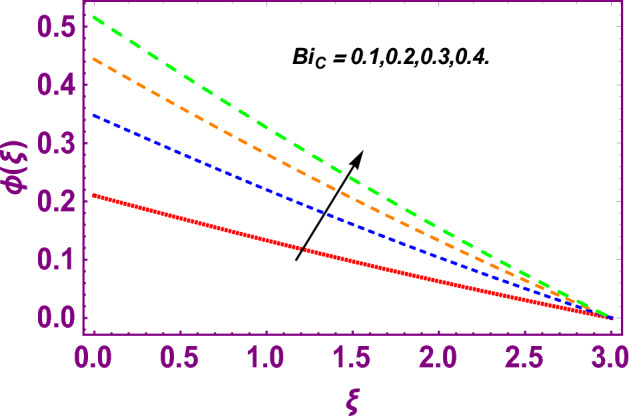
Influence of concentration Biot number upon $\phi \left( \xi \right)$.

**Figure 12:**
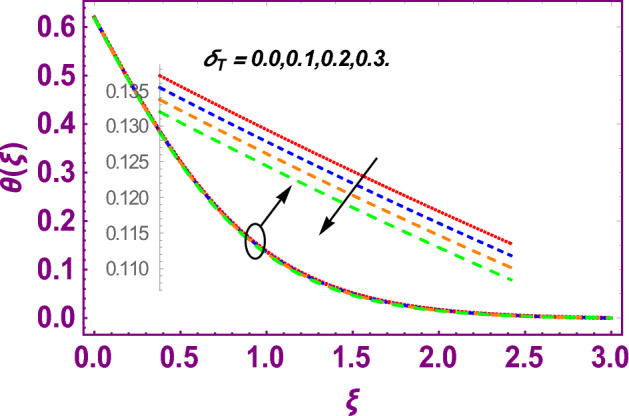
Effect of $\delta_{T}$ on $\theta \left( \xi \right)$.

**Figure 13:**
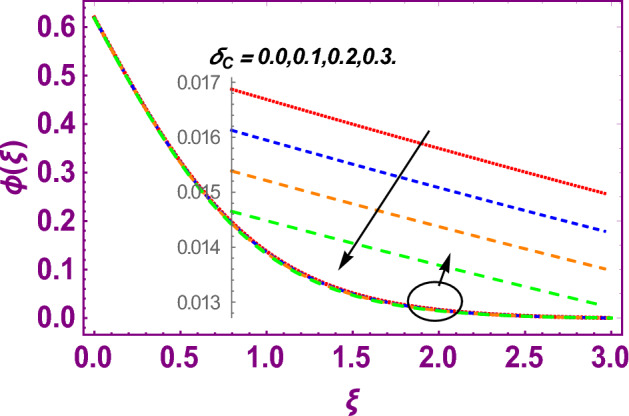
Effect of $\delta_{C}$ on $\phi \left( \xi \right)$.

**Figure 14:**
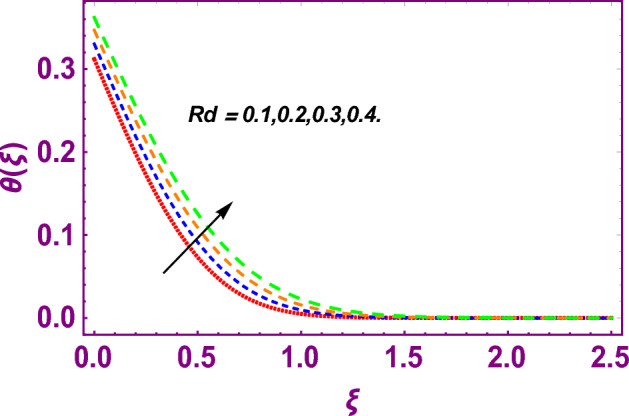
Effect of $Rd$ on $\theta \left( \xi \right)$.

**Figure 15:**
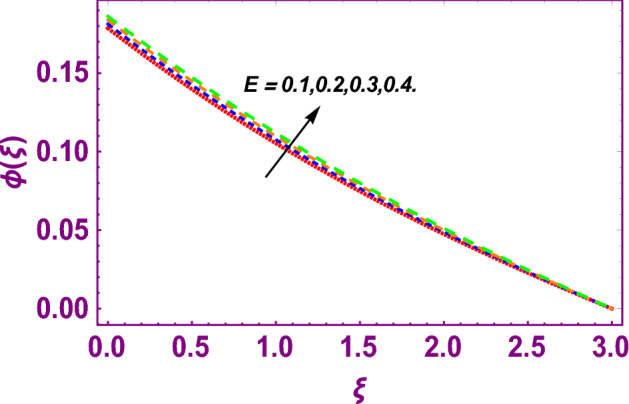
Effect of $E$ on $\phi \left( \xi \right)$.

**Figure 16:**
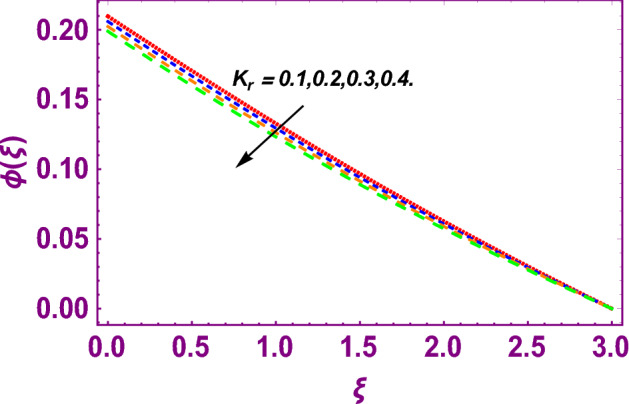
Effect of $K_{r}$ on $\phi \left( \xi \right)$.

This work addresses the 3D electromagnetohydrodynamic nanofluid flow past a bi-directional Riga plate with slip and convective boundary conditions. Nanofluid is composed of zinc-oxide nanoparticles which are suspended in water. The CC model for heat and mass flux is used in a flow problem. Moreover, thermal radiation is incorporated in energy equation and the effects of *E* and *K*_*r*_ are employed in concentration equation. The numerical investigation of the flow is incorporated with the help of NDSolve technique. Figure 1a shows the Flow chart. Figure 2 presents the influence of volumetric fraction $\Pi$ of $ZnO$ nanoparticles on velocity profile $f^{\prime}\left( \xi \right)$. Since with a growing $\Pi$, the density of fluid augments due to which the fluid becomes denser. In this phenomenon fluid experienced more friction due to resistive force in the direction of motion. As a result, velocity profile $f^{\prime}\left( \xi \right)$ reduces. A similar impact of $\Pi$ is depicted against velocity profile $g^{\prime}\left( \xi \right)$ as shown in Fig. 3. Figure 4 shows the impact of $\Pi$ on $\theta \left( \xi \right)$. The increasing $\Pi$ escalates $\theta \left( \xi \right)$. Physically, the thermal conductivity of ${\mathrm{ZnO}}{-}{\mathrm{H}}_{2} {\mathrm{O}}$ enhances with the increasing $\Pi$ which results the enhancement in the thermal boundary layer thickness and temperature profile. Therefore, the increasing $\Pi$ increases $\theta \left( \xi \right)$. Figure 5 shows the effect of modified Hartmann number $H$ on velocity profile $f^{\prime}\left( \xi \right)$. The increasing $H$ increases $f^{\prime}\left( \xi \right)$. The momentum boundary layer thickness also increases with the increasing $H$. Because the modified Hartmann number has bigger values, the external electric field is increased, maximizing the velocity distribution $f^{\prime}\left( \xi \right)$. Similar impacts were found in^39,48,49^. The influences of stretching ratio factor $\lambda$ on $f^{\prime}\left( \xi \right)$ and $g^{\prime}\left( \xi \right)$ are depicted in Figs. 6 and 7. It is observed that the increasing $\lambda$ reduces the velocity profile $f^{\prime}\left( \xi \right)$, while increases the velocity profile $g^{\prime}\left( \xi \right)$. The reason is that the stretching parameter has direct relation with the stretching velocity constant along *y*-direction and inverse relation with the stretching velocity constant along *x*-direction. Therefore, a decreasing impact is found for $f^{\prime}\left( \xi \right)$, while an increasing impact found for $g^{\prime}\left( \xi \right)$. Similar impacts were found in^48,49^. Figures 8 and 9 depict the influence of slip parameter $\alpha$ on $f^{\prime}\left( \xi \right)$ and $g^{\prime}\left( \xi \right)$. It has observed that $f^{\prime}\left( \xi \right)$ and $g^{\prime}\left( \xi \right)$ declines with higher values of $\alpha$. Actually, when the values of $\alpha$ become higher, then some of the stretching velocities transformed to the fluid particles that results in retardation of fluid motion in all directions. Due to the decline in momentum boundary layer thicknesses the velocities $f^{\prime}\left( \xi \right)$ and $g^{\prime}\left( \xi \right)$ are declined. Figure 10 depicts that with augmentation in thermal Biot number $Bi_{T}$ there is a minimization in thermal resistance amongst nanoparticles at the surface of a Riga plate. Hence the thermal distribution at the Riga plate upsurge due to augmentation in $Bi_{T}$, that results in maximizing the thermal flow profile as portrayed in Fig. 10. Similarly the growth in the values of concentration Biot number leads to augmentation in concentration distribution as shown in Fig. 11. Figure 12 presents the influence of thermal relaxation factor $\delta_{T}$ on thermal flow profile. It is noticed from this figure that the upsurge in $\delta_{T}$ retards thermal characteristics. Actually, this parameter is employed as an indicator for estimating the quantity of time that is required for heat transmission from one zone to another one of the same materials. When $\delta_{T}$ is enhanced then more time is required for thermal flow from one zone to another, hence there is a retarding behavior in heat transportation. Therefore, the increasing $\delta_{T}$ decreases the thermal profile. The influence of mass relaxation factor $\delta_{C}$ on concentration profile is depicted in Fig. 13. The factor $\delta_{C}$ is actually an indicator used for estimation of amount of time needed for mass diffusions from higher to lower concentration zone. The augmentation in $\delta_{C}$ indicates that more time is required for transmission of mass from one region to another that ultimately declines the concentration of nanofluid. Figure 14 depicts the influence of radiation factor $Rd$ on thermal profile. It is obvious from the figure that heat transmission jumps up with higher values of $Rd$. Physically this can be interpreted as the growth in $Rd$ augments the heat energy amongst nanoparticles closed to the Riga plate. In this process the charge on these nanoparticles augments, that ultimately grows up the thermal profile. Figure 15 portrays the influence of activation energy factor $E$ on concentration profile. For higher values of $E$, a bulk of molecules that entails small quantity of energy supports diffusions of mass. Therefore, higher values of $E$ results a growth in the concentration profile. The influence of chemical reaction parameter $K_{r}$ on concentration profile is depicted in Fig. 16. Intensifying values of $K_{r}$ results the retardation of mass diffusivity that weakens the thickness of concentration boundary layer thickness. Hence, higher values of $K_{r}$ causes a decline in concentration profile. Using variations in different parameters the results of current investigation have been compared with established results in the literature. In Tables 2 and 3, a comparison is carried out for $- f^{\prime\prime}\left( {\xi = 0} \right)$ and $- g^{\prime\prime}\left( {\xi = 0} \right)$ with the results of Iqbal et al.^50^, Khan et al.^51^ and Makinde and Aziz^52^ for different values of stretching ratio parameter, while keeping other parameters zero. A fine agreement of current results with those established in literature has been found. In Table 4, it has noticed that the values of $C_{sx}$ and $C_{sy}$ have been retarded with augmentation in the values of solid volume fraction, modified Hartmann number, stretching ratio and slip factors. The numerical impacts of radiation parameter, solid volume fraction and thermal Biot number on Nusselt number have been described. In Table 5, it is noticed that the resistive force to fluid motion has been increased for augmentation of these stated parameters. Therefore, the values of Nusselt number has been upsurge due to augmenting values of radiation parameter, solid volume fraction and thermal Biot number.

**Table 2:** Comparison of current results of $- f^{\prime\prime}\left( {\xi = 0} \right)$ with previously reported results, when $\Pi = 0.0$.

$\lambda$	Iqbal et al.^50^	Khan et al.^51^	Makinde and Aziz^52^	Present results
0.00	1.0000	1.0000	1.0000	1.00000
0.25	1.048813	1.048813	1.048812	1.05428
0.50	1.093097	1.093095	1.093095	1.09731
0.75	1.134485	1.134485	1.134485	1.13782
1.00	1.173720	1.173721	1.173721	1.17641

**Table 3:** Comparison of current results of $- g^{\prime\prime}\left( {\xi = 0} \right)$ with previously reported results, when $\Pi = 0.0$.

$\lambda$	Iqbal et al.^50^	Khan et al.^51^	Makinde and Aziz^52^	Present results
0.00	0.000000	0.000000	0.000000	0.000000
0.25	0.194564	0.194564	0.194564	0.197344
0.50	0.465205	0.465205	0.465205	0.468343
0.75	0.794622	0.794622	0.794622	0.797595
1.00	1.173720	1.173720	1.173720	1.176410

**Table 4:** Impacts of the embedded parameters on $C_{sx}$ and $C_{sy}$.

$\Pi$	$\alpha$	$H$	$\lambda$	$C_{sx}$	$C_{sy}$
0.01	0.1	0.9	0.5	0.817115	0.409982
0.03				0.795033	0.394869
0.04				0.782609	0.386907
0.06				0.755505	0.370360
	0.2			0.663296	0.330800
	0.3			0.592753	0.299782
	0.4			0.536768	0.274672
		0.2		0.589796	0.272797
		0.5		0.566982	0.273606
		0.7		0.551849	0.274141
			0.2	0.518715	0.095370
			0.4	0.531060	0.211124
			0.6	0.542225	0.341353

**Table 5:** Impacts of the embedded parameters on $Nu$.

$\Pi$	$Rd$	$Bi_{T}$	$Nu$
0.01	0.1	0.1	0.104389
0.03			0.105025
0.04			0.106852
0.06			0.108529
	0.2		0.111480
	0.3		0.119405
	0.4		0.127304
		0.2	0.197853
		0.3	0.284146
		0.4	0.363343

## Conclusion

This work addresses the three-dimensional electromagnetohydrodynamic flow of a water-based zinc-oxide nanofluid over a bi-directional Riga plate with velocity slips and thermal convective boundary conditions. The Cattaneo–Christov heat and mass flux model is taken into consideration in order to analyze the thermal and concentration profiles. Moreover, thermal radiation effect is consideration in energy equation and the effects of Arrhenius activation energy and chemical reaction are taken in concentration equation. A suitable set of similarity variables is used to transform the equations that governed the flow problem, into dimension free form. During this process some parameters are created and influences of these factors on flow profiles are discussed. A numerical technique called NDSolve is considered for the solution of the nanofluid flow problem. The results showed that higher solid volume fraction and slip parameter have reduced velocities profiles and the increasing solid volume fraction and thermal Biot number have increased the temperature profile. Additionally, the concentration Biot number has increased the concentration profile. The modified Hartmann number has significantly increased the velocity profile. Dual impacts in velocity profiles along primary and secondary direction has been observed due to stretching ratio parameter. The increasing thermal and mass relaxation factors have augmented the temperature and concentration profiles, respectively. The concentration profile is increased due to chemical reaction parameter while increased due to activation energy parameter. A comparison of current results has been carried with a fine agreement amongst current and established results. The Nusselt number is upsurge due to augmenting values of radiation parameter, solid volume fraction and thermal Biot number.

## Data Availability

All data used in this manuscript have been presented within the article.

## List of symbols

$$a_{0}$$: Electrode size
$$a$$,$$b$$: Constants
$Bi_{T}$: Thermal Biot number
$Bi_{C}$: Concentration Biot number
$$C$$: Concentration
$$C_{p}$$: Specific heat
$D_{B}$: Coefficient of Brownian diffusivity
$E$: Activation energy parameter
$h_{T}$: Convective heat transfer coefficient
$H$: Modified Hartmann number
$h_{C}$: Convective mass transfer coefficient
$$j_{0}$$: Current density
$$k$$: Thermal conductivity
$K_{r}$: Chemical reaction factor
$k^{*}$: Mean absorption coefficient
$$m$$: Power index
$$M_{0} \left( x \right)$$: Magnetization strength
$$Sc$$: Schmidt number
$$T$$: Temperature
$$u,v,w$$: Velocity components

## Greek letters

$\lambda_{E}$: Dimensional thermal relaxation time
$\delta_{T}$: Dimensionless thermal relaxation parameter
$\lambda$: Stretching ratio parameter
$\lambda_{C}$: Dimensional mass relaxation time
$\delta_{C}$: Dimensionless mass relaxation parameter
$$\mu$$: Dynamic viscosity
$$\rho$$: Density
$$\alpha$$: Slip parameter
$$\alpha_{0}$$: Slip length
$\Pi$: Volume fraction of nanoparticle
$\sigma^{*}$: Stefan–Boltzmann constant
$\beta$: EMHD material parameter

## Subscripts

$$nf$$: Nanofluid
$$f$$: Fluid
$$n$$: Nanoparticle
$w$: At the surface
$$\infty$$: Free stream

## Abbreviations

$${\mathrm{ZnO}}$$: Zinc oxide
$${\mathrm{H}}_{2} {\mathrm{O}}$$: Water
EMHD: Electro-magneto-hydrodynamic
